# Music in Noise: Neural Correlates Underlying Noise Tolerance in Music-Induced Emotion

**DOI:** 10.1093/texcom/tgab061

**Published:** 2021-10-13

**Authors:** Shota Murai, Ae Na Yang, Shizuko Hiryu, Kohta I Kobayasi

**Affiliations:** Graduate School of Life and Medical Sciences, Doshisha University, 1-3 Miyakodani, Tatara, Kyotanabe, Kyoto 610-0321, Japan; Graduate School of Life and Medical Sciences, Doshisha University, 1-3 Miyakodani, Tatara, Kyotanabe, Kyoto 610-0321, Japan; Graduate School of Life and Medical Sciences, Doshisha University, 1-3 Miyakodani, Tatara, Kyotanabe, Kyoto 610-0321, Japan; Graduate School of Life and Medical Sciences, Doshisha University, 1-3 Miyakodani, Tatara, Kyotanabe, Kyoto 610-0321, Japan

**Keywords:** auditory–motor, chills, functional magnetic resonance imaging, striatum

## Abstract

Music can be experienced in various acoustic qualities. In this study, we investigated how the acoustic quality of the music can influence strong emotional experiences, such as musical chills, and the neural activity. The music’s acoustic quality was controlled by adding noise to musical pieces. Participants listened to clear and noisy musical pieces and pressed a button when they experienced chills. We estimated neural activity in response to chills under both clear and noisy conditions using functional magnetic resonance imaging (fMRI). The behavioral data revealed that compared with the clear condition, the noisy condition dramatically decreased the number of chills and duration of chills. The fMRI results showed that under both noisy and clear conditions the supplementary motor area, insula, and superior temporal gyrus were similarly activated when participants experienced chills. The involvement of these brain regions may be crucial for music-induced emotional processes under the noisy as well as the clear condition. In addition, we found a decrease in the activation of the right superior temporal sulcus when experiencing chills under the noisy condition, which suggests that music-induced emotional processing is sensitive to acoustic quality.

## Introduction

Music provides emotional experiences in everyday life. Sometimes, these can be particularly strong and accompanied by the sensation of “chills,” which are used as indicators of intense emotional experiences related to music (Goldstein 1980; Sloboda 1991; Panksepp 1995; Grewe et al. 2009; Salimpoor et al. 2009). Chills denote pleasant physical sensations, such as a shiver or goose bumps, that accompany the increased activity of the sympathetic nervous system. The majority of experiences of chills occur at the highest moment of pleasure. Behavioral experiments assess the experience of chills via self-report using button responses and demonstrate that the experience of chills occurred from a few to approximately 10 times per musical piece and lasted from one to several tens of seconds (Rickard 2004; Craig 2005; Salimpoor et al. 2011; Mori and Iwanaga 2017; Ferreri et al. 2019; Bannister 2020; Klepzig et al. 2020; Laeng et al. 2021). Several factors are known to influence the intense emotional experience as one listens to music, such as familiarity with a musical piece, personal traits (Grewe et al. 2007), and acoustic patterns, such as unexpected changes in melody and loudness and the entry of vocals (Sloboda 1991; Grewe et al. 2007). In particular, temporal changes in spectral features influence the probability that chills are experienced (Nagel et al. 2008). Thus, perceptual processes that analyze spectrotemporal features and predict dynamic changes in sound patterns are expected to underlie the occurrence of intense emotional experiences.

Brain imaging studies have demonstrated the neural basis of chills, including the superior temporal region (i.e., superior temporal sulcus/gyrus, STS/STG), supplementary motor area (SMA) insula, anterior cingulate cortex (ACC), putamen, and caudate (Blood and Zatorre 2001; Salimpoor et al. 2011; Martínez-Molina et al. 2016; Sachs et al. 2016; Klepzig et al. 2020). The bilateral superior temporal regions show music-selective neural responses (Fedorenko et al. 2012; Boebinger et al. 2021). In particular, spectral features, such as musical chord and melodic contour, are processed in the right STS (Klein and Zatorre 2011; Lee et al. 2011). The SMA and putamen as well as the superior temporal region are related to the analysis of musical temporal patterns (Ferrandez et al. 2003; Wiener et al. 2010; Kung et al. 2013; Herrmann et al. 2014). The caudate is involved in the anticipation of pleasure derived from music (Salimpoor et al. 2011). It has also been reported that several of the regions mentioned above can be involved in the inhibition of the experience of chills. Patients with lesions on the insula were shown to lack the experience of chills and enjoyment of music (Griffiths et al. 2004; Grunkina et al. 2017). Furthermore, individuals with musical anhedonia were shown to have weak functional connectivity between the right superior temporal region and striatum (Martínez-Molina et al. 2016), while a group less sensitive to chills also had weak structural connectivity between the right posterior superior temporal region, insula, and frontal region (Sachs et al. 2016).

However, the effect of the degradation of acoustic quality on the experience of chills and the modulation of the neural activation pattern of brain regions associated with chills remain unclear. Typically, music is enjoyed under a noisy condition, such as at concerts or in clubs, which degrades acoustic features as a trigger of chills. Such a listening situation would make it difficult to understand the acoustic features and alter the processes related to chills. In the case of understanding acoustically degraded speech, additional cognitive resources are required for auditory processing compared with when signals are acoustically clear (Davis and Johnsrude 2003; Eckert et al. 2016; Peelle 2017). In addition, the noisy timbre in vocalization and music have been associated with primary emotions with low valence and high arousal, such as anger and fear (Wallmark et al. 2018). In collaboration, listening under the noisy condition would modulate the auditory and emotional processes for chills compared with listening under the clear condition.

Therefore, in the present study, we examined how acoustic quality modulates music-induced emotional responses by investigating similarities and differences in the processes of chills between clear and noisy conditions. To create a noisy listening situation, we added noise to musical pieces. The participants listened to “clear music” and “noisy music,” and we collected data on their reports whether they experienced chills. We measured neural activity in response to the experience of chills using functional magnetic resonance imaging (fMRI). By adding noise to musical pieces, we aimed to explore the effect of acoustic quality on musical emotional processing. We hypothesized that adding noise would reduce the chance of chills, and even if chills did occur, the noisy condition would shorten their duration. Additionally, the superior temporal and motor regions associated with temporal processes during music perception eliciting emotion would show robust activation during experiencing chills under the noisy condition where spectral details in musical pieces were deteriorated while the temporal patterns were preserved. Moreover, we also hypothesized that the noisy condition would reduce the activation of the right STS, sensitive to musical spectral features within the superior temporal regions.

## Materials and Methods

### Participants

The participants were 19 right-handed healthy individuals (12 females and 7 males; age range: 21–24 years) who can provide music pieces that previously elicited the experience of chills. All participants provided informed written consent prior to taking part in the experiment. Data from one participant were excluded due to aberrant behavioral responses (i.e., duration of chills exceeded 3 standard deviations from the average duration), suggesting a failure to correctly complete the tasks. The study was approved by the Ethics Committee of Doshisha University, Japan. All participants were monolingual speakers of Japanese with normal hearing (thresholds of <20 dB HL at 125–8000 Hz, according to audiometry; Rion AA-77A, Tokyo, Japan).

### Stimuli

**Figure 1:**
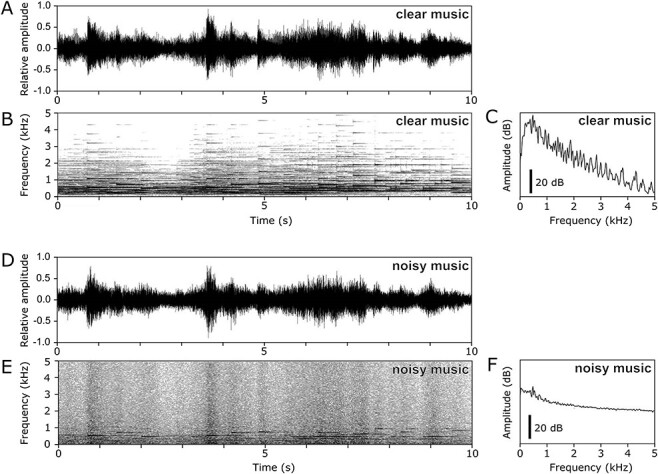
Excerpts of the musical stimuli. (A) Waveform, (B) spectrogram, and (C) a power spectrum of an excerpt of clear music. (D) Waveform, (E) spectrogram, and (F) a power spectrum of an excerpt of noisy music.

The participants were asked to provide four musical pieces that had previously evoked chills in them, with no restrictions on genre. These pieces were used to examine the emotional responses elicited, independent of a specific genre (Salimpoor et al. 2009). Details on various genres and acoustical characteristics of music pieces were provided in the Supplementary Material  Table S1. We also selected two other pieces of music. One was from the same genre or artist as the music provided by the participant, while the other was classical music (“Clair de Lune,” composed by Claude Debussy). Thereby, 5 out of 6 pieces varied across participants, and whether the musical pieces had lyrics depended on the music participants provided. These 6 musical pieces were referred to as “clear” music. We also prepared 6 pieces of “noisy” music created from the 6 pieces of clear music. In total, each of 12 pieces was presented once for one single participant. The noisy music consisted of music and pink noise (with a similar amplitude envelope as the clear music) in a sound intensity ratio of music: pink noise = 1:2 (Fig. 1). The amplitude envelope derived from clear music was extracted by half-wave rectification and low-pass filtering at 5 Hz and multiplied by pink noise. The resultant modulated pink noise and clear music were summed to generate noisy music. The auditory stimuli were processed using Matlab (MathWorks, Inc., Natick, MA, USA). A mean duration per piece was 267 s. Notably, clear music was acoustically clearer than noisy music; however, both clear and noisy music was presented over scanner noise. The participants heard 12 pieces of music presented at 90–95 dB SPL, while using earplugs (mean sound attenuation of 30 dB) to reduce the scanner noise. The musical stimuli were presented via MRI-compatible headphones (AS3000K, Kiyohara Optics, Inc., Tokyo, Japan) during fMRI scanning.

### Procedure

**Figure 2:**
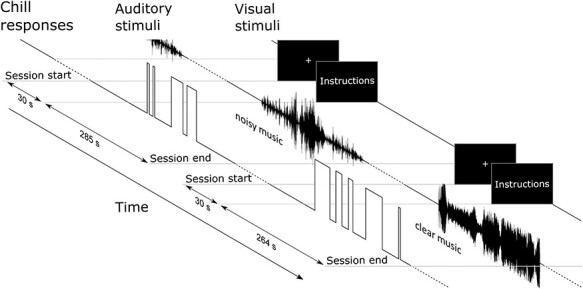
Schematic representation of the experiment.

The participants listened to 12 pieces of music via headphones while lying down in the MRI scanner. One piece of music was presented per session (Fig. 2), resulting in 12 sessions in total. In each session, a piece of music was presented after a rest period of 30 s. The duration of sessions depended on music pieces, then the mean duration of sessions was 303 s. The order in which the clear and noisy music sessions were presented was randomized. The noisy music sessions were interleaved with clear music sessions. To clarify the duration of their experience of chills in real time, participants reported whether they had chill experience (i.e., chills) or nonchill experience (neutral) in response to the music, using two buttons marked “chills” or “neutral” on an input device (HHSC-1 × 4-D, Current Designs, Inc., Philadelphia, PA, USA) held in their left hand. They were asked to press and hold the appropriate button while listening to the music. At the end of a musical piece, participants were asked to rate how the music made them feel on a 5-point scale (arousal: 1 = low arousal to 5 = high arousal; valence: 1 = unpleasant to 5 = pleasant). Due to technical problems, ratings were not collected from 6 participants. The experiment was programmed with the Presentation software package (Neurobehavioral Systems, Inc., Albany, CA, USA).

### Behavioral Analysis

To evaluate the differences between participants’ emotional experiences while listing to noisy music versus clear music, we counted the number of musical pieces during which participants had reported experiencing chills and the number of chills experienced within a musical piece. We also used the duration participants experienced chills while listening to a musical piece. The duration of chills was defined as the period during which a participant reported experiencing chills by holding down the appropriate button. The number of musical pieces, the number of chills, and the duration of chills were compared between acoustic qualities (noisy and clear). Moreover, we focused on chills that occurred at similar time points for a musical piece in the clear and noisy conditions (common chills) to avoid psychoacoustic differences as potential confounders. Typically, chills occur at different times during a musical piece between the clear and noisy conditions, and such differences in timing could induce different psychoacoustic processes related to the experience of chills. Common chills were defined as chills that occurred within an interval of 10 s between acoustic qualities (noisy and clear). The proportions of common chills in the total number of chills were then computed, and the durations of common chills were also compared between acoustic qualities. The statistical analyses of behavioral data were performed in R (R Core Team 2020) with the packages rstatix (Kassambara 2021) for two-tailed Wilcoxon signed-rank tests and with the package ARTool (Kay et al. 2021) for nonparametric ANOVA.

### fMRI Data Acquisition and Analysis

We recorded the T1-weighted structural images (echo time [TE] = 4 ms, repetition time [TR] = 9.4 ms, flip angle = 8, field of view [FOV] = 256 × 256 mm, 192 slices, inversion time [TI] = 1013 ms, voxel size = 1 × 1 × 1 mm) and echo-planar whole-brain volumes (30 slices; TR = 3000 ms; TE: 50 ms; 4 mm thickness with a 1 mm gap; transverse; FOV: 192 × 192 mm; matrix size: 64 × 64; fractional anisotropy [FA]: 90°) as functional imaging data on a 1.5-tesla MRI scanner (Echelon Vega, Hitachi Medical, Chiba, Japan), according to a previously reported protocol (Okuya et al. 2017).

To identify distinct brain activity in response to either clear or noisy music within participants, we analyzed the data for 14 participants (10 females and 4 males) who reported chills during the noisy music sessions. Because the primary focus of this study was to examine the robustness of emotional processes against acoustic differences, we analyzed the fMRI experimental sessions in which participants had chills while listening to music under both clear and noisy conditions. The data were analyzed using SPM12 software (Wellcome Trust Centre for Neuroimaging, University College London, London, UK; http://www.fil.ion.ucl.ac.uk/spm/) for realignment, spatially normalization to an EPI template in the Montreal Neurological Institute (MNI) space, and smoothing with an 8 mm full-width at half-maximum Gaussian kernel and statistical analyses, which were performed using a general linear model (GLM). At the single-subject level, the model included 4 regressors: (1) chills (onset of chill button press) under the clear condition, (2) chills under the noisy condition, (3) neutral responses (onset of nonchill button press) under the clear condition, and (4) neutral responses under the noisy conditions. We additionally entered a nuisance regressor that was sound intensity (root mean squares) using the MIR toolbox (Lartillot et al. 2008). These regressors were convolved with the canonical hemodynamic response function. Head movement parameters from realignment corrections and block regressors were also added as regressors of no interest. A first-order autoregressive model corrected the serial correlations among scans. A brain activation map was estimated based on a regressor containing different numbers of behavioral responses. We analyzed the contrast of chills or neutral responses against the implicit baseline (i.e., a baseline not explicitly modeled in a GLM and estimated as the average of the residual time series within a session). At the group-level, the participants’ contrast maps were subjected to random-effect analyses while considering variance across individuals. We employed a full-factorial design and conducted a null-conjunction of chills under the clear condition and noisy condition to examine brain regions that were significantly active in both conditions.

To quantify differences in chill-related activity between clear and noisy conditions, a second GLM analysis was performed. To avoid the psychoacoustical differences as potential confounders, the second GLM included regressors for common chills and corresponding neutral responses and a nuisance regressor for all other responses. We compared activity of common chills experienced while listening to clear musical pieces with that of common chills experienced while listening to noisy musical pieces. A group-level analysis was conducted using one-sample *t*-tests.

Brain activation was examined with *t*-contrasts at a voxel-level threshold of *P* < 0.001 and a cluster-level family-wise error (cFWE)-corrected threshold of *P* < 0.05 for multiple comparisons at the whole-brain level. To avoid motor confounders in brain activation due to button press, we used an exclusive mask of activity associated with neutral responses (i.e., nonchill button press) obtained by using a null-conjunction of neutral in clear music and noisy music at a voxel-level uncorrected threshold of *P* < 0.05 with cFWE-corrected *P* < 0.05. Anatomical labels were automatically obtained using an automated anatomical labeling (AAL) toolbox (Tzourio-Mazoyer et al. 2002) and Anatomy toolbox (Eickhoff et al. 2005). fMRI data were displayed using NeuroElf (version 1.1; http://neuroelf.net/) and MRIcronGL software (https://www.mccauslandcenter.sc.edu/mricrogl/).

In addition, we evaluated neural activity associated with chills and neutral responses under the clear and noisy conditions by region of interest (ROI) analysis using the leave-one-participant-out approach to avoid nonindependence bias (Esterman et al. 2010). First, for one participant, the peak coordinate from the contrast between common chills under the clear and noisy conditions at the group-level analysis, which left the participant, was determined within an anatomical mask of the superior temporal region based on the AAL atlas (Tzourio-Mazoyer et al. 2002). The ROI was then created as an 8-mm radius sphere centered on the peak coordinate. Using the leave-one-participant-out ROI, parameter estimates were extracted for the participant. Building ROIs and extracting parameter estimates were performed using MarsBaR toolbox (http://marsbar.sourceforge.net/).

## Results

### Behavioral Results

**Figure 3:**
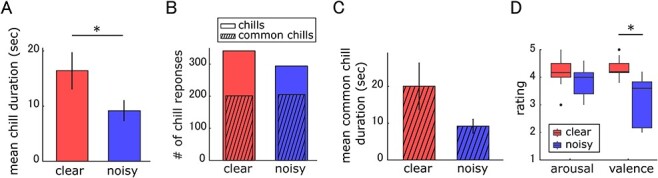
Behavioral results. (A) Mean chill duration across participants (*n* = 14). (B) Total number of chill responses across participants (*n* = 14). Hatched area represents number of common chills, defined as observed chills with similar timing in the clear and noisy music conditions. Please see the main text for details. (C) Mean common chills duration across participants (*n* = 14). (D) Boxplots of scores for arousal and valence ratings (*n* = 9). Error bars indicate standard error of the mean. ^*^P < 0.05, Wilcoxon signed-rank test.

The behavioral data showed that 18 participants reported chills under the clear music conditions, whereas 14 participants of them also reported chills under the noisy music conditions. Subjective ratings were acquired from 9 out of 14 participants. To focus on within-participant effects of acoustic quality (clear and noisy music), the subsequent analysis was based on data from the 14 participants who experienced chills in both conditions and from the musical pieces during which they experienced chills. Data from these 14 participants showed that the mean number (±standard deviation) of musical pieces during which they experienced chills was 5.9 ± 0.4 under the clear condition and 4.4 ± 1.7 under the noisy condition. They experienced chills for a significantly smaller number of musical pieces under the noisy condition compared with the clear condition (*P* = 0.008, *r* = 0.78, Wilcoxon signed-rank test). The mean number of chills per musical piece decreased under the noisy condition (5.5 ± 3.4), relative to the clear condition (6.5 ± 3.4), but the difference was not significant (*P* = 0.07, *r* = 0.49, Wilcoxon signed-rank test). The mean duration of chills was 16.2 ± 12.8 s in the clear condition and 9.4 ± 7.9 s in the noisy condition. Chill duration under the noisy condition was significantly shorter than under the clear condition (*P* = 0.01, *r* = 0.66, Wilcoxon signed-rank test; Fig. 3A). The proportions of common chills in the total number of chills were 58.9% and 69.7% in the clear and noisy conditions, respectively (Fig. 3B). Mean duration of common chills was 20.1 ± 22.7 s in the clear condition and 9.2 ± 6.7 s in the noisy condition. The mean duration of common chills in the noisy condition was shorter than that in the clear condition; however, the difference was not significant (*P* = 0.06, Wilcoxon signed-rank test; Fig. 3C).

The results of participants’ subjective ratings of emotional arousal and valence are shown in Figure 3D. The mean arousal across participants was 4.2 ± 0.6 and 3.8 ± 0.5 for clear and noisy condition, respectively, and the statistical analyses indicated that the difference was not significant (*P* = 0.173, *r* = 0.47, Wilcoxon signed-rank test; Fig. 3D). The mean valence ratings across participants were 4.3 ± 0.4 and 3.0 ± 0.9 for the clear and noisy conditions, respectively. The results indicated that valence significantly differed between the clear and noisy conditions (*P* = 0.004, *r* = 0.89, Wilcoxon signed-rank test; Fig. 3D). We further conducted nonparametric ANOVA with the factors of acoustic quality (clear and noisy conditions) and emotion dimension (arousal and valence) on subjective ratings using the aligned rank transform method (Wobbrock et al. 2011). The results demonstrated the significant main effect of acoustic quality (*F*(1, 172) = 30.7, *P* < 0.001, *η_p_*^2^ = 0.15) and the significant main effect of emotion dimension (*F*(1, 172) = 5.73, *P* = 0.018, *η_p_*^2^ = 0.03). In addition, a significant interaction was observed between acoustic quality and emotion dimension (*F*(1, 172) = 6.20, *P* = 0.014, *η_p_^2^* = 0.04), which indicates that valence was more sensitive to noisiness than arousal was.

We further assessed whether the acoustic quality (i.e., noisy or clear) can have different impacts on musical experience depending on music genre. Pop and classical music were compared as the sample sizes of other genres were small. The following parameters were analyzed: chill duration, number of chills, and emotional rating for all genres are reported in Tables S2 and S3; Supplementary Materials. We conducted nonparametric ANOVA with the factors of acoustic quality (clear and noisy conditions) and genre (pop and classic) on behavioral data to examine interaction effects. The findings indicated that no interaction exists between acoustic quality and genre for chill duration (*F*(1, 72) = 0.54, *P* = 0.465, *η_p_*^2^ = 0.007), number of chills (*F*(1, 72) = 0.61, *P* = 0.44, *η_p_*^2^ = 0.008), valence rating (*F*(1, 56) = 0.44, *P* = 0.511, *η_p_*^2^ = 0.008), and arousal rating (*F*(1, 56) = 0.05, *P* = 0.832, *η_p_*^2^ = 0.001). These results did not indicate that acoustic quality could lead to differential impacts on emotional experiences for each genre.

**Figure 4:**
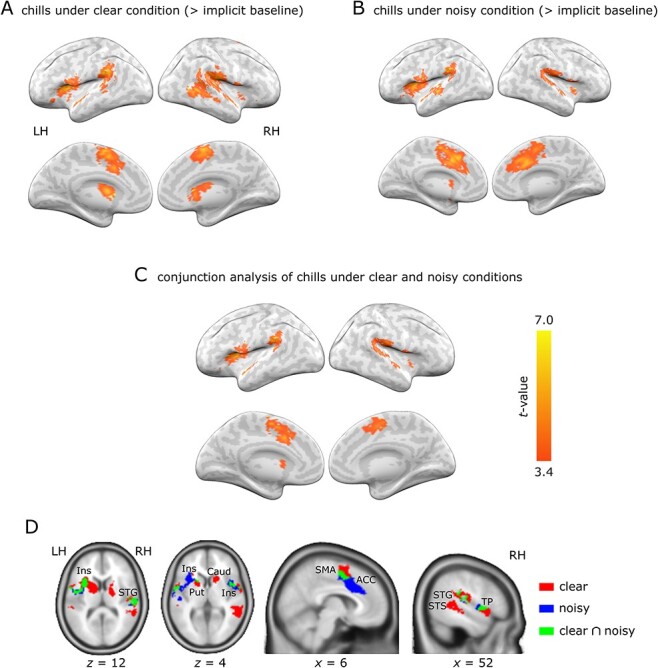
Results of the whole-brain analysis. (A, B) Brain activity associated with chills under the clear and noisy conditions relative to implicit baseline. (C) Results of conjunction analysis showed activated brain regions shared by chills under the clear condition and chills under the noisy condition. (D) Axial and sagittal sections. *n* = 14. *P* < 0.05 cluster-level FWE corrected. Clear; chills under the clear condition; noisy, chills under the noisy condition; RH, right hemisphere; LH, left hemisphere; *x, z* = MNI coordinates.

### Brain Activity Related to Chills while Listening to Clear and Noisy Music

We conducted whole-brain analyses to examine the blood oxygenation level-dependent signal amplitude that occurred when the participants reported chills under clear and noisy conditions. Chills experienced while listening to clear music increased brain activity in several regions relative to implicit baseline, including the precentral gyrus, supramarginal gyrus, middle temporal gyrus, STS/STG, temporal pole, left superior medial frontal area, SMA, middle cingulate gyrus, insula, putamen, and caudate (Fig. 4A, D, Table 1), which was consistent with previous studies (Blood and Zatorre 2001; Salimpoor et al. 2011). We analyzed neural responses to chills during listening to noisy music. Increased activity was observed in the precentral gyrus, supramarginal gyrus, middle temporal gyrus, STG, putamen, insula, and ACC (Fig. 4B, D, Table 1). Furthermore, conjunction analysis was performed to identify shared brain regions related to chills between the clear and noisy music conditions. This analysis revealed overlapping activity for chills in the clear and noisy conditions in the right precentral gyrus, supramarginal gyrus, left SMA, temporal pole, bilateral STG, and insula (Fig. 4C, D, Table 1). The conjunction analysis did not show activity in the putamen, we found activation peaks within the putamen for chills under the clear condition and chills under the noisy condition, respectively (Table 1). To avoid potential confounders between chills under the clear and noisy conditions, conjunction analysis was also performed between common chills under clear and noisy conditions. We did not find significant activation, although activation was observed in brain regions similar to the conjunction analysis on all chill events at a liberal threshold (*P* < 0.05 uncorrected) (see Supplementary Material for details).

**Table 1:** Clusters of brain regions showing significant activation

		MNI Coordinates			Region		
Extent	*t*-value	*x*	*y*	*z*	side	AAL	Anatomy
Chills under the clear condition							
444	6.89	−64	−40	30	L	Supramarginal gyrus	Supramarginal gyrus
444	6.01	−56	−40	26	L	Supramarginal gyrus	Superior temporal gyrus
1223	6.87	−34	12	12	L	Insula	Insula lobe
1223	6.19	−18	10	6	L	Putamen	Putamen
1002	6.24	−2	2	62	L	Supplementary motor area	Posterior–medial frontal
1002	6.22	−10	12	38	L	Middle cingulate gyrus	MCC
1002	5.89	−6	−2	54	L		Posterior–medial frontal
1292	6.22	50	−22	18	R	Rolandic operculum	Rolandic operculum
1292	5.96	58	−42	4	R	Middle temporal gyrus	Middle temporal gyrus
1292	5.69	46	−26	−6	R	Location not in atlas	Location not in atlas
357	5.55	18	−4	12	R	Thalamus	Pallidum
357	4.97	16	18	4	R	Caudate	Caudate nucleus
357	3.93	18	6	10	R		Location not in atlas
376	5.22	56	14	−4	R	Inferior frontal, opercular part	Temporal pole
376	5.21	50	6	0	R	Rolandic operculum	Insula lobe
376	5.14	42	10	2	R	Insula	Insula lobe
Chills under the noisy condition							
1217	7.24	−34	12	12	L	Insula	Insula lobe
1217	6.49	−50	−20	−2	L	Middle temporal gyrus	Middle temporal gyrus
1217	5.74	−22	12	−6	L	Putamen	Location not in atlas
1887	6.57	−10	16	40	L	Middle cingulate gyrus	MCC
1887	6.10	−6	−4	54	L	Supplementary motor area	Posterior–medial frontal
1887	5.74	8	12	34	R	Middle cingulate gyrus	ACC
183	6.47	−64	−40	30	L	Supramarginal gyrus	Supramarginal gyrus
183	6.10	−58	−36	24	L		Superior temporal gyrus
241	6.03	42	10	2	R	Insula	Insula lobe
241	5.24	50	4	2	R	Rolandic operculum	Insula lobe
241	4.10	60	−2	2	R	Superior temporal gyrus	Superior temporal gyrus
343	4.95	62	−16	16	R	Postcentral gyrus	Superior temporal gyrus
343	4.71	60	−28	16	R	Superior temporal gyrus	Superior temporal gyrus
Conjunction analysis of chills under the clear and noisy conditions							
498	6.87	−34	12	12	L	Insula	Insula lobe
498	5.63	−56	8	−2	L	Temporal pole: superior temporal gyrus	Temporal pole
498	5.01	−32	22	8	L		Insula lobe
183	6.47	−64	−40	30	L	Supramarginal gyrus	Supramarginal gyrus
183	5.76	−58	−36	24	L		Superior temporal gyrus
510	6.22	−10	12	38	L	Middle cingulate gyrus	MCC
510	5.89	−6	−2	54	L	Supplementary motor area	Posterior–medial frontal
510	4.84	6	0	52	R	Supplementary motor area	Posterior–medial frontal
157	5.14	42	10	2	R	Insula	Insula lobe
157	5.05	50	4	0	R	Rolandic operculum	Insula lobe
157	4.10	60	−2	2	R	Superior temporal gyrus	Superior temporal gyrus
289	4.95	62	−16	16	R	Postcentral gyrus	Superior temporal gyrus
289	4.47	60	−28	16	R	Superior temporal gyrus	Superior temporal gyrus
289	4.27	52	−30	28	R	Supramarginal gyrus	Rolandic operculum
Common chills under the clear condition > common chills under the noisy condition							
101	6.38	46	−42	10	R	Middle temporal gyrus	Middle temporal gyrus
101	4.93	40	−48	8	R		Location not in atlas
101	4.68	52	−44	16	R	Superior temporal gyrus	Superior temporal gyrus

The table shows the local maxima up to three separated by more than 8 mm per cluster (SPM default). Regions were labeled using the automated anatomical labeling (AAL) and Anatomy toolbox. If a cluster had the same combinations of labels obtained from the AAL and Anatomy toolbox, only the peak showing a higher *t*-value was displayed. *x*, *y*, and *z* = Montreal Neurological Institute (MNI) coordinates in the left–right, anterior–posterior, and inferior–superior dimensions, respectively. *n* = 14. *P* < 0.05 cluster-level FWE corrected.

### Brain Activity in the Right Superior Temporal Sulcus Induced by Chills during Clear Music

**Figure 5:**
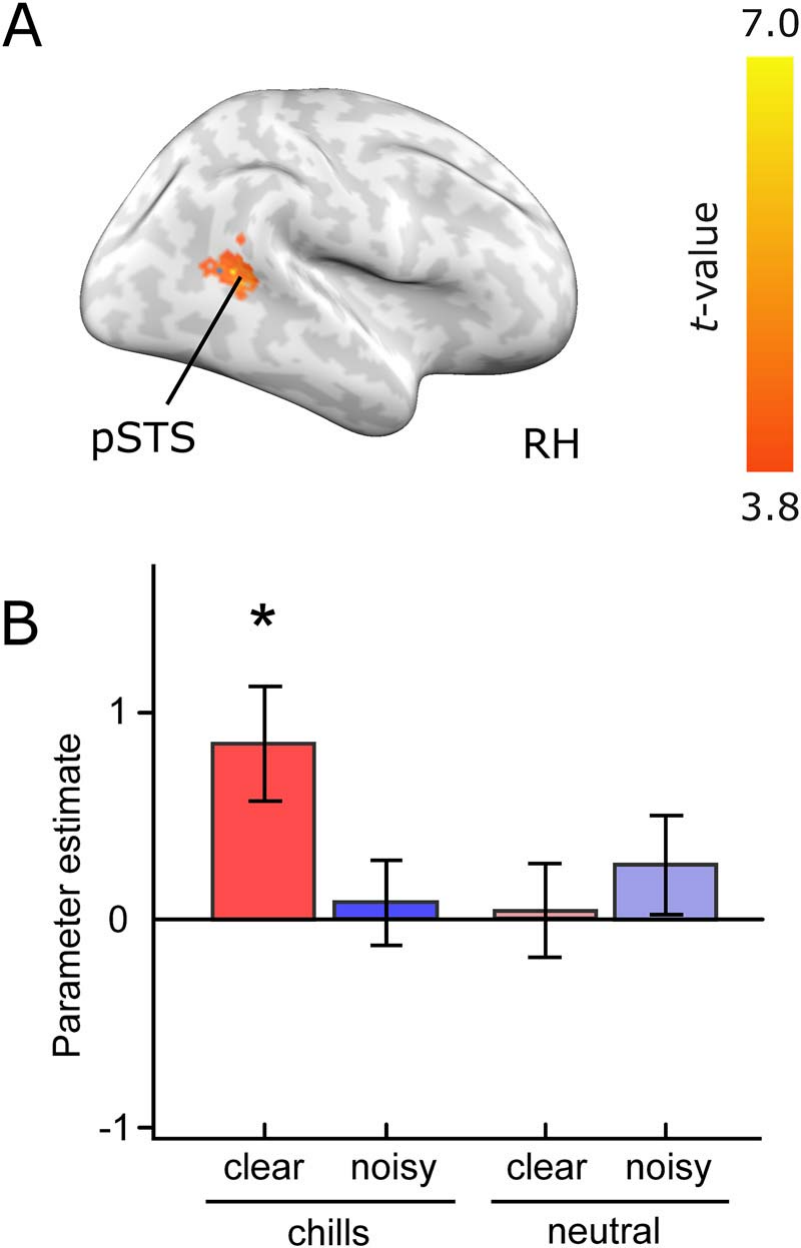
(A) Increased activity for common chills under the clear compared to the noisy condition. *P* < 0.05; cluster-level FWE corrected. (B) Parameter estimates for each condition. Error bars indicate the standard errors of the mean. The significant activation against zero is displayed by asterisks (^*^P < 0.05, Wilcoxon signed-rank test with Bonferroni correction). *n* = 14. RH: right hemisphere; pSTS: posterior superior temporal sulcus; clear: chills under the clear condition; and noisy: chills under the noisy condition.

To analyze effects of acoustic quality on neural activity for chills while avoiding the effect of specific musical features on chills, we compared brain activity related to common chills between the clear and noisy music conditions. Common chills in the clear music conditions compared with those in the noisy music were associated with activity in the right posterior superior and middle temporal gyri including the STS (Fig. 5A, and Table 1). This means that the region around the right posterior STS showed chill-related activity sensitive to acoustic quality. Contrastingly, there was no significantly greater activation associated with common chills in the noisy music condition when compared with common chills in the clear music condition.

In this comparison based on common chills, however, chill-irrelevant responses to acoustic quality could be confounded. To address this potential confound, we further analyzed activity in the right STS for chill and neutral responses under the clear and noisy conditions using the leave-one-participant-out ROI analysis (Fig. 5B). The mean ROI coordinates across participants was [44.7, −40.9, 9.4] at the bottom of the superior temporal region approximately equivalent to the STS. The results indicated that the activity was significantly responsive to chills under the clear conditions (*P* = 0.007, Wilcoxon signed-rank test) but not to the three other conditions (*P* > 0.05, Wilcoxon signed-rank test).

## Discussion

The present study investigated the relationship between acoustic differences (clear and noisy musical pieces) and the neural activity elicited by strong emotional responses to music. In this manner, it aims to reveal the modulating effect of acoustic quality on music-induced emotional processes. Under the clear condition, neural activity in the temporal regions, insula, medial frontal regions, and striatum was positively correlated with chills, whereas the activation pattern observed was consistent with the findings of previous studies (Fig. 4A and D; Table 1; Blood and Zatorre 2001; Salimpoor et al. 2011; Klepzig et al. 2020). In contrast, the noisy condition decreased the opportunities for participants to experience chills and led to shortened chill durations. Moreover, the noisy and clear conditions activated partially overlapping regions (Fig. 4C and D; Table 1). This finding indicates that music-induced emotional processes are robust against the degradation of acoustic quality.

In the clear condition, activations were found in the posterior part of the right STS and caudate, while the same regions did not show activation under the noisy condition (Fig. 4B, D and Table 1). The right STS are known to be sensitive to spectral features like musical chord and melodic contour (Klein and Zatorre 2011; Lee et al. 2011), and the caudate is thought to be involved in the anticipation of musical pleasure (Salimpoor et al. 2011). The noisy musical pieces lacked spectral details (Fig. 1C, F), and the decreases in spectrum information might have interfered with the acoustic process in the temporal regions, thereby impairing the anticipatory processes mediated in the caudate. In a previous study that manipulated the spectral and temporal structures in music, the caudate exhibited decreased activation due to spectral instead of temporal disruption (Kim et al. 2019). In addition, decreased neural activity under the noisy condition corresponded well with our findings regarding behavioral responses, in that valence ratings also decreased under the noisy condition. To assess the effects of spectral degradation under the noisy condition on neural activation in the right STS and caudate, we further conducted correlation analyses between fMRI activation and acoustic characteristics. The result pointed to a weak correlation between key clarity, which indicates spectral details in musical pieces and chill-related activity in the right superior temporal region (*β* = 4.69, *P* = 0.091; see Supplementary Material). However, the right caudate did not exhibit a significant correlation with key clarity (*β* = 9.96, *P* = 0.198). Deactivation in the right STS may influence the caudate processes of chill through cortico-striatal connectivity. Thus, further studies are required to reveal the relationship between spectral degradation and connectivity.

Furthermore, we compared activation between common chills epochs under noisy and clear conditions. The analysis then found a significantly different activation in a more restricted area, the posterior part of the right STS (Fig. 5 and Table 1). Because comparing common chills allows us to directly analyze the effects of acoustic quality, these results suggest that the posterior part of the STS could be involved in acoustic processing related to chills.

Chills under the noisy condition showed more extended activation in the left anterior insula and anterior of the cingulate cortex relative to chills under the clear condition (Fig. 4B, D). The insula and ACC have been reported to work together to facilitate cognitive resources (i.e., attentional and working memory) in salient events (Menon and Uddin 2010) and support the recognition of speech sounds under difficult listening conditions (Eckert et al. 2016). Hence, these regions may also contribute to the perception of musical pieces under difficult listening conditions, and activation of the insula and ACC in the noisy condition of our experiment may reflect complementary roles in perceiving musical pieces and experiencing emotions. Notably, the activation for chills under the clear condition was estimated from the imbalanced designs between the clear and noisy conditions at the single-subject level. Although controlling for the number of chill experiences is difficult, further studies with balanced data are required to elucidate whether the degradation of acoustic quality increases chill-related neural responses associated with complementary mechanisms for auditory and emotional processing.

Finally, the bilateral STG, insula, SMA, and putamen showed overlapping activations between the clear and noisy conditions (Fig. 4C, D and Table 1). We assumed that these regions were involved in core processes of chills and strong emotional experiences, because their activities were robust to acoustic quality. Previous behavioral studies have revealed that unexpected temporal changes in musical patterns (e.g., timing of changes in melody, loudness, and the entry of a voice) could be possible triggers for chills (Grewe et al. 2007), thus suggesting temporal analysis of music, such as segregation and prediction, is a critical process of chills. Many previous studies have shown that analysis of temporal patterns in music is related to the superior temporal regions, SMA, and putamen (Ferrandez et al. 2003; Wiener et al. 2010; Kung et al. 2013; Herrmann et al. 2014). Therefore, activation of the superior temporal regions, SMA, and putamen as reported in the present study could be attributed to the analysis of temporal patterns in music during strong emotional experiences. In addition to chill-related temporal-analysis networks, we found overlapping activations in the insula and temporal pole. Several clinical studies have shown that these areas have an essential role in the emotional aspect of experiencing chills. For example, a previous study reported that a patient with a lesion on the insula lost his ability to enjoy music, even though he had no impairment in his ability to perceive sound (Griffiths et al. 2004). Similarly, another patient was reported to have lost the ability to have the bodily response of chills due to a lesion on the insula (Grunkina et al. 2017). Temporal pole atrophy has also been reported to result in a lack experiencing emotional arousal from music (Hsieh et al. 2012). Thus, common activation of the insula and temporal pole might contribute to the robustness of the emotional aspect of chills against acoustic noise.

The present study had a limitation. Physiological measures such as skin conductance and heart rate were not measured simultaneously with MRI scanning; although arousal induced by chills has been shown to be associated with these physiological responses (Rickard 2004; Salimpoor et al. 2009; Mori and Iwanaga 2017). As we did in the present study, all previous fMRI studies estimated the experience of chills based on subjective participant reports; however, they also measured the physiological indices simultaneously during scanning (Salimpoor et al. 2011; Martínez-Molina et al. 2016; Grunkina et al. 2017; Klepzig et al. 2020). Thus, future studies should examine the effect of noisy conditions on subjective responses and neural activity along with additional measurements of physiological indices. Our analysis is further limited by the number of the experience of chills. Except for common chills analysis, the chill-related activation was estimated from the GLM containing different numbers of chill experiences between the clear and noisy conditions at the single-subject level analysis. Further studies with balanced data are required to assess the similarities or differences in chill-related neural responses between the clear and noisy conditions and to elucidate how the musical emotional processing is interfered with or complemented under the acoustically degraded condition.

In conclusion, our findings suggest that noise may reduce the duration of chills and affect the activation of higher auditory and reward systems. However, brain regions associated with emotion and temporal processes during music perception showed robust activation in the noisy condition. These could serve as a neurophysiological underpinning by which music induces pleasurable states, even in noisy listening situation, and help to shed light on the relationship between sound quality and music-induced emotions.

## Supplementary Material

Supplementary_material_2021Sep25th_tgab061
