# Supplementary material for: Music in Noise: Neural Correlates Underlying Noise Tolerance in Music-Induced Emotion
Source: Cereb Cortex Commun. 2021 Oct 13;2(4):tgab061. doi: 10.1093/texcom/tgab061 (PMC8564766; doi:10.1093/texcom/tgab061)
Supplement: Supplementary_material_2021Sep25th_tgab061 [file supplementary_material_2021sep25th_tgab061.docx]

Supplementary Material

Music in noise: Neural correlates underlying noise tolerance in music-induced emotion

## Properties of music pieces

Table S1 showed music genres and acoustic characteristics of music pieces used in behavioral and fMRI analyses. According to a previous study examining relationships between musical chills and genre (Nusbaum and Silvia 2011), music pieces were categorized into genres used in a musical genre study (Rentfrow and Gosling 2003). Music genres of the pieces were categorized by a majority vote of three participants who did not participate the behavioral and fMRI experiments. Only one music pieces was referred to an online music store, as the piece was categorized as three different genres by the participants. Acoustic characteristics for each music genre were analyzed using the MIR toolbox (Lartillot et al. 2008).

Table S1. Stimulus properties.

|  |  | Clear | | | | |  | Noisy | | |
| --- | --- | --- | --- | --- | --- | --- | --- | --- | --- | --- |
|  | N | Tempo |  | Key  Clarity | Pulse  Clarity | Event  Density |  | Key  Clarity | Pulse  Clarity | Event Density |
| Pop | 22 | 121.5  (16.0) |  | 0.63  (0.05) | 0.29  (0.10) | 2.94  (0.66) |  | 0.59 (0.06) | 0.25 (0.08) | 2.45 (0.58) |
| Classical | 16 | 105.9  (9.8) |  | 0.67  (0.03) | 0.18  (0.04) | 0.69  (0.51) |  | 0.62 (0.04) | 0.15 (0.03) | 0.55 (0.41) |
| Rock | 6 | 126.9  (16.8) |  | 0.58  (0.04) | 0.27  (0.05) | 3.59  (0.33) |  | 0.53 (0.06) | 0.22 (0.03) | 3.09 (0.21) |
| Sound track | 5 | 119.9  (8.1) |  | 0.61  (0.03) | 0.18  (0.02) | 0.96  (0.35) |  | 0.58 (0.04) | 0.17 (0.02) | 0.73 (0.27) |
| Electronica/dance | 4 | 127.2  (8.0) |  | 0.61  (0.09) | 0.36  (0.07) | 3.15  (1.35) |  | 0.58 (0.08) | 0.29 (0.05) | 2.68 (1.42) |
| Religious | 3 | 106.5  (13.9) |  | 0.66  (0.02) | 0.20  (0.02) | 0.79  (0.25) |  | 0.63 (0.03) | 0.17 (0.02) | 0.62 (0.21) |
| Folk | 2 | 119.9  (14.4) |  | 0.64  (0.01) | 0.21  (0.02) | 1.51  (0.17) |  | 0.54 (0.07) | 0.19 (0.01) | 1.40 (0.13) |
| Alternative | 1 | 113.0 |  | 0.61 | 0.66 | 2.35 |  | 0.58 | 0.61 | 2.21 |
| Jazz | 1 | 131.5 |  | 0.43 | 0.41 | 3.99 |  | 0.42 | 0.34 | 3.05 |
| Rap/hip-hop | 1 | 148.2 |  | 0.63 | 0.38 | 2.84 |  | 0.61 | 0.35 | 2.28 |

Mean values (± standard deviation, where applicable) of acoustic characteristics across music pieces in each genre. N, the number of music pieces.

## Behavioral results for each genre

Table S2. Behavioral results of chill responses for each genre.

|  |  | Clear | |  | Noisy | |
| --- | --- | --- | --- | --- | --- | --- |
|  | N | # of Chills | Chill Duration (s) |  | # of Chills | Chill Duration (s) |
| Pop | 22 | 8.4 (6.1) | 13.7 (10.6) |  | 6.5 (6.0) | 8.7 (7.3) |
| Classical | 16 | 7.5 (6.8) | 12.3 (17.2) |  | 7.2 (7.5) | 6.9 (6.8) |
| Rock | 6 | 6.3 (3.4) | 15.4 (17.3) |  | 3.3 (1.6) | 10.5 (5.6) |
| Sound track | 5 | 6.0 (2.5) | 12.1 (6.5) |  | 5.4 (5.3) | 2.8 (1.8) |
| Electronica/dance | 4 | 10.3 (9.2) | 12.96 (7.9) |  | 9.5 (9.8) | 17.0 (11.5) |
| Religious | 3 | 2.7 (1.2) | 39.64 (27.7) |  | 1.7 (0.6) | 19.6 (10.7) |
| Folk | 2 | 6.0 (2.8) | 8.5 (7.0) |  | 4.5 (3.5) | 9.8 (12.6) |
| Jazz | 1 | 5 | 14 |  | 4 | 8.48 |
| Rap/hip-hop | 1 | 2 | 31.42 |  | 1 | 23.34 |
| Alternative | 1 | 4 | 25.43 |  | 9 | 14.74 |

Mean values (± standard deviation, where applicable) of chill responses across music pieces in each genre. N, the number of music pieces.

Table S3. Emotional ratings for each genre.

|  |  | Clear | |  | Noisy | |
| --- | --- | --- | --- | --- | --- | --- |
|  | N | Arousal | Valence |  | Arousal | Valence |
| Pop | 18 | 4.7 (1.0) | 4.4 (0.9) |  | 4.2 (0.7) | 2.9 (1.2) |
| Classical | 12 | 3.6 (1.4) | 4.3 (0.7) |  | 3.0 (1.0) | 2.7 (1.5) |
| Rock | 5 | 4.4 (0.9) | 4.0 (0) |  | 3.6 (0.9) | 3.6 (0.9) |
| Religious | 3 | 3.7 (1.5) | 4.0 (1.7) |  | 4.3 (1.2) | 2.7 (2.1) |
| Folk | 2 | 3.0 (0) | 4.0 (0) |  | 4.0 (1.4) | 3.5 (0.7) |
| Sound track | 2 | 4.5 (0.7) | 5.0 (0) |  | 4.0 (1.4) | 3.0 (1.4) |
| Electronica/dance | 1 | 5.0 | 4.0 |  | 5.0 | 5.0 |
| Rap/hip-hop | 1 | 3.0 | 4.0 |  | 3.0 | 4.0 |

Mean values (± standard deviation, where applicable) of emotional ratings across music pieces in each genre. N, the number of music pieces.

## Conjunction analysis between common chills in clear and noisy conditions


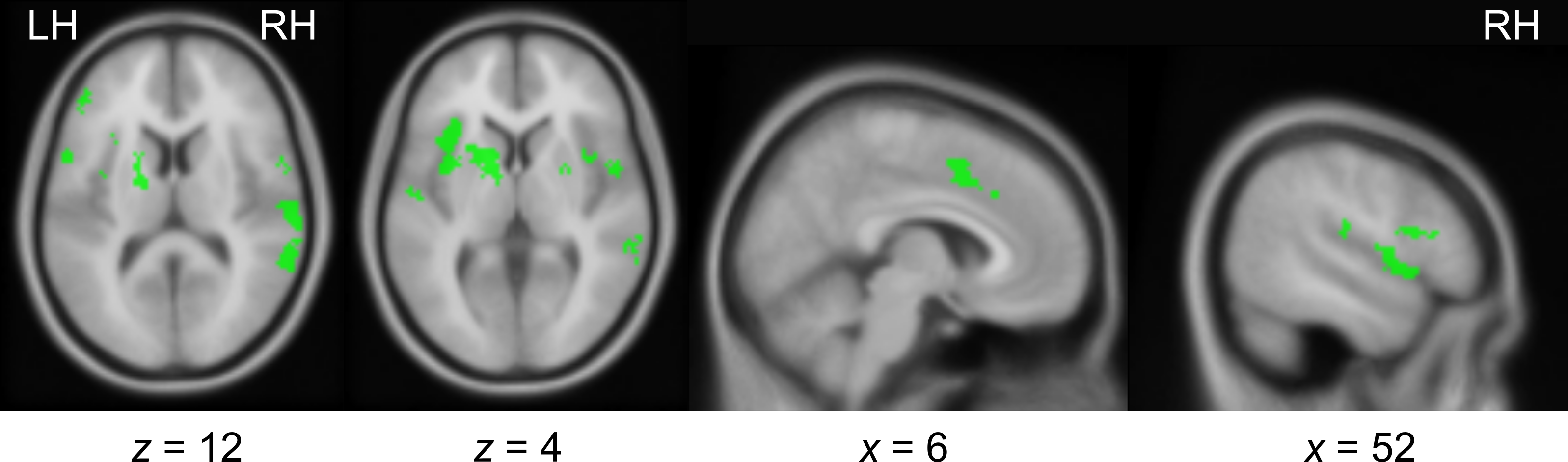


Figure S1. fMRI results obtained from conjunction analysis showing activated brain regions shared by chills under the clear condition and chills under the noisy condition (green). n = 14. *p* < 0.05 uncorrected for multiple comparisons. RH, right hemisphere; LH, left hemisphere; x, z = MNI coordinates.

## Correlation analysis between fMRI activation and acoustic characteristics

Analysis of the effect of acoustic characteristics (key clarity or pulse clarity) on the fMRI activation for chills under the noisy condition in the right pSTS or the right caudate was conducted. The region of interests (ROI) was created based on the AAL atlas (Tzourio-Mazoyer et al. 2002). The right pSTS was defined as a region within the superior temporal area and located at a posterior position relative to the most posterior coordinate of the Heschl’s gyrus. The “lme4” package in R was used to test hypotheses with linear mixed-effect models. Acoustic characteristics were set as a fixed effect. Participants were set as a random effect. As a result, there was no significant effect of acoustic characteristics (*p* > 0.05); however, only main effect of key clarity demonstrated a weak effect (*p* > 0. 1, *β* = 4.69), indicating that the right pSTS activity for chills increased with increasing key clarity.

# References

Lartillot O, Toiviainen P, Eerola T. 2008. A Matlab Toolbox for Music Information Retrieval. In: Data Analysis, Machine Learning and Applications. p. 261–268.

Tzourio-Mazoyer N, Landeau B, Papathanassiou D, Crivello F, Etard O, Delcroix N, Mazoyer B, Joliot M. 2002. Automated anatomical labeling of activations in SPM using a macroscopic anatomical parcellation of the MNI MRI single-subject brain. Neuroimage. 15:273–289.
